# Dietary Supplementation with Whole-Fat or Defatted Antarctic Krill Powder Improves the Growth Performance, Body Coloration,  and Immune Capability of Red–White Koi Carp (*Cyprinus carpio* var. koi)

**DOI:** 10.3390/ani15111561

**Published:** 2025-05-27

**Authors:** Hongmei Song, Yixin Liang, Yexin Yang, Chao Liu, Yi Liu, Xidong Mu, Xuejie Wang

**Affiliations:** Key Laboratory of Tropical and Subtropical Fishery Resources Application and Cultivation, Ministry of Agriculture and Rural Affairs, Pearl River Fisheries Research Institute, Chinese Academy of Fishery Sciences, Guangzhou 510380, China; liangyixin1218@163.com (Y.L.); yangyexin@163.com (Y.Y.); liuchao@prfri.ac.cn (C.L.); seetohrb@163.com (Y.L.); muxd1019@163.com (X.M.); aqwang2@163.com (X.W.)

**Keywords:** koi, Antarctic krill powder, growth performance, body color, immune capability

## Abstract

This study was conducted to investigate the effects of replacing fish meal with either whole-fat or defatted krill powder on the growth, body color, immunity, and related gene expression of red–white koi carp. A total of 630 red–white koi carp with an initial body mass of 13.5 ± 0.05 g were randomly divided into seven groups with three replicates per group and 30 fish per replicate. The control group was fed a basic diet (C0). The other six diets were supplemented with different levels of whole krill meal or defatted krill meal as replacements (10% whole fat, 20% whole fat, 30% whole fat, 10% defatted, 20% defatted, and 30% defatted) in the experimental groups, named W10, W20, W30, D10, D20, and D30, respectively, for a total duration of 60 days. The growth, body color, immunity and gene expression indexes were measured in the koi after completion. The results indicate the following. (1) Compared with C0, the experimental groups of koi showed a significant increase in the specific growth rate (SGR) (*p* < 0.05), while the hepatosomatic index (HSI) and viscerosomatic ratio (VSI) decreased. Additionally, there was a significant increase in the relative expression level of insulin-like growth factor (*IGF-1*) in both the liver and muscle (*p* < 0.05). (2) The experimental groups of koi carp exhibited a significant increase in the carotenoid content in the scales and skin, as well as an elevated relative expression level of the tyrosinase (*TYR*) gene in the muscle (*p* < 0.05). (3) The lysozyme (LZM) and superoxide dismutase (SOD) activities were significantly increased in the experimental groups compared to C0 (*p* < 0.05). Additionally, the SOD activity was significantly higher in the defatted groups than in the whole-fat groups (*p* < 0.05). Furthermore, the liver alkaline phosphatase (AKP) activity was significantly lower in the D20 and D30 groups compared to the other five groups. (4) The expression level of *LPL* was significantly lower in the liver and muscle of the whole-fat group compared to the defatted group (*p* < 0.05). Tissue section observation revealed that the hepatocytes in the W20 and W30 groups exhibited a reduced size and an increased lipid droplet count, while the vacuolar degeneration of the hepatocytes increased in the D30 group. In conclusion, replacing fish meal with whole-fat or defatted krill powder or defatted Antarctic krill meal significantly improves the growth performance, body color, and immunity of red–white koi carp. However, excessive addition of krill meal can easily cause liver damage. The recommended replacement level for whole krill powder is 20% to 30%, while defatted krill powder should be replaced at a level of 10% to 20%.

## Simple Summary

Replacing fish meal with varying levels of whole-fat or defatted Antarctic krill meal can offer the following advantages: (1) enhanced SGR and WGR, and reduced HSI and VSI, thereby improving the growth performance of red-and-white carp; (2) increased carotenoid content in the scales and skin, along with enhanced expression of *TYR* for improved body coloration; (3) augmented lysozyme and superoxide dismutase enzyme activities, with better antioxidant effects observed in the defatted krill meal replacement group; and (4) substitution of whole-fat krill meal significantly downregulates the expression of *LPL*. Meanwhile, elevated levels of whole-fat replacement groups lead to a reduction in the liver AKP enzyme activity, as well as an increase in the number of lipid droplets. However, the defatted replacement groups at high levels exhibit a significant increase in the vacuolated degeneration of liver cells and various degrees of liver injury. It is recommended that the replacement level of whole-fat krill meal in compound feed, with a fish meal supplemental level of 500 g/kg, should be between 20 and 30%, while the replacement level of defatted krill meal should be between 10 and 20%.

## 1. Introduction

Koi (*Cyprinus carpio* var. koi) is a freshwater fish endemic to subtropical and temperate zones. It is a famous ornamental fish with high economic value, named for its vibrant and diverse colors as well as its ever-changing markings. Koi have a strong and vigorous body shape, a lively yet steady swimming posture, and an extremely long lifespan (averaging 60 to 70 years). It is often praised as the “lucky fish”, a “living jewel in water”, and an “artwork that can swim”. The body shape, pattern changes, and color quality of koi are the main factors that affect their economic and aesthetic value [1]. These are also important morphological characteristics considered in breeding programs. Among the various determinants impacting the body coloration of a particular fish species, such as the light exposure, genetic inheritance, physiological processes, and diet composition, feeding with coloring additives is the most effective way to enhance the body coloration of koi carp. Fish cannot synthesize pigments themselves; however, most freshwater fish species like koi have the ability to convert zeaxanthin [2], beta-carotene, lutein, and other carotenoids into astaxanthin. Moreover, they can effectively accumulate astaxanthin in their bodies to enhance their body coloration [3,4]. Therefore, it becomes essential for the sustainable growth of koi aquaculture to develop a compound feed for koi that both satisfies their nutritional needs and enhances their body color.

With the continuous development of the aquaculture industry, fish meal has become the primary high-quality protein source in aquatic feed due to its nutritional value, palatability, and ease of digestion and absorption by aquatic animals. Consequently, the increasing demand for fish meal results in a continuing price increase. Therefore, it is necessary for the current aquatic feed industry to find an alternative protein source that can replace fish meal [5]. Currently, research primarily focuses on plant-based options, such as rapeseed meal, cottonseed meal and peanut meal, which are widely available and affordable. However, “plant” protein sources often have drawbacks, including poor palatability, amino acid imbalances, high cellulose content, and difficult digestion [6]. Animal protein like blood meal, meat meal, and feather meal can partially substitute for fish meal; however, their utilization is currently limited due to concerns about bovine spongiform encephalopathy and avian influenza [7].

Antarctic krill is one of the species with the largest biological reserves in the world’s oceans, boasting a balanced amino acid and fatty acid nutritional profile, as well as strong palatability and no anti-nutritional factors [8]. Processed Antarctic krill powder can be used as a new high-quality protein source to add to aquatic feed, offering great potential for research on fish meal replacement [9]. The commercially available Antarctic krill powder includes defatted krill powder and whole krill powder. Defatted krill powder is a by-product of extracting krill oil from whole krill powder, which not only includes the muscle component of Antarctic krill but also the exoskeleton component. Studies have shown that defatted Antarctic krill powder contains a high proportion of chitin and fluorine, which affects its addition ratio in feed [10]. The Antarctic krill oil in the whole krill powder is rich in unsaturated fatty acids, which makes it more prone to oxidation in the powder and unsuitable for long-term storage [8,11]. Additionally, there are certain differences in the amino acid composition between whole krill powder and defatted krill powder [12]. Currently, both whole-fat and defatted Antarctic krill powders have been investigated in terms of feeding various fish species, including in clownfish (*Amphiprion latezonatus*) [13], rainbow trout (*Oncorhynchus mykiss*) [7], and Atlantic salmon (*Salmo salar*) [4], as well as *Platichthys stellates* [14], *Scophthalmus maximus* [15], *Tilapia nilotica* [16], and others. Defatted krill meal, a novel protein source, has also been studied for its use in aquaculture feed for species such as *Verasper variegatus* [13] and *Sparus macrocephalus* [17]; however, relatively less research has been conducted on its application [12]. Furthermore, Antarctic krill powder is abundant in astaxanthin, with a content ranging from 15 to 200 mg/kg. This can also be used as an additive to enhance the body color of fish [18]. However, there is still a lack of references regarding the replacement of fish meal with whole-fat or defatted krill powder for regulating the growth performance and body color of koi carp. Therefore, this study aimed to investigate the effects of replacing fish meal with different proportions of whole or defatted krill meal on the growth, body color, liver biochemical indexes, and expression of genes related to growth and body color in red-and-white koi carp. The objective was to determine the optimal level of krill meal as a substitute for fish meal and provide a reference for future studies on alternative animal protein sources.

## 2. Materials and Methods

### 2.1. Experimental Fish and Feeding Management

A total of 630 red–white koi, with an initial body mass of 13.5 ± 0.05 g and consistent initial body color at the age of 4 months from the same parent stock, were selected as the experimental subjects, and the experiment was conducted at the ornamental fish base of the Pearl River Fisheries Research Institute, Chinese Academy of Fishery Sciences (Guangzhou, China). The experimental fish were randomly divided into 7 groups, consisting of one control group and six experimental groups. Each group had 3 replicates, with each replicate containing 30 fish. The fish were placed in 21 glass aquariums measuring 120 cm × 60 cm× 50 cm and fed a basal diet for 14 days prior to the start of the experiment. During the experiment, feeding was conducted twice daily at 7:00 a.m. and at 2:00 p.m. The daily feeding amount was approximately 3% of the fish body mass, adjusted incrementally as the fish grew. The water temperature was maintained at 27 ± 1 °C, while the pH level was kept within the range of 7.6 to 7.8. Partial water changes (one-third of the total volume) were performed three times per week. Continuous aeration was provided throughout the experiment to ensure adequate oxygenation.

### 2.2. Experimental Feed

The feed formula and nutrition level are presented in Table 1. Imported fish meal, soybean protein, and wheat flour were utilized as the main sources of protein, while fish oil and soybean oil served as fat sources for preparing the basic feed. Whole krill meal (with full fat) or defatted krill meal (without fat) was added to the basic feed. The fish meal replacement levels for the seven groups of fish were 0%, 10% with full fat, 20% with full fat, 30% with full fat, 10% defatted, 20% defatted, and 30% defatted, respectively. These levels were recorded as C0 (basic feed), W10, W20, W30, D10, D20, and D30. All the raw materials used for feeding were crushed using a grinder and passed through an 80-mesh screen. After completing the natural air-drying process, the extruded pellet feeds with a diameter of 2 mm were prepared and stored at −20 °C.

### 2.3. Sample Collection

The experimental period lasted for 60 days. Prior to and following the experiment, the experimental fish were fasted for 24 h, and their body length and mass were measured. At the conclusion of the culture experiment, the number of experimental fish in each group was counted, and six randomly selected fish from each replicate were anesthetized. Blood was then drawn from their tail veins, which was left at a temperature of 4 °C for 6 h before being centrifuged. The resulting supernatant was stored at −80 °C for later use. Approximately 0.05 g of scales and skin was collected to determine the carotenoid content. The liver and visceral mass were dissected and weighed to calculate the ratio between the liver mass and the total viscera mass. From each replicate, two random fish had their livers and muscles taken for RNA extraction by placing them in RNAlater solution. Additionally, a small amount of liver tissue was extracted for section preparation by placing it in a centrifuge tube with paraformaldehyde.

### 2.4. Index Measurement

#### 2.4.1. Growth Index Measurement

The specific growth rate (SGR), weight gain rate (WGR), survival rate (SR), condition factor (CF), hepatosomatic index (HSI) and viscerosomatic index (VSI) of the fish were calculated using the following formulas.

Specific growth rate, SGR, % = 100 × (lnWt − lnW0)/t

Weight gain rate, WGR, % = 100 × (Wt − W0)/W0

Survival rate, SR, % = 100 × survival individuals/total individuals

Condition factor, CF, g/cm^3^ = 100 × Wt/L^3^

Hepatosomatic index (HSI, %) = 100 × total liver mass (g)/experimental fish body mass (g)

Viscerosomatic index (VSI, %) = 100 × visceral mass (g)/experimental fish body mass (g)

In the above formula, W0 represents the initial body mass of the experimental fish (g), Wt represents the final body mass of the experimental fish (g), t represents the number of experimental days (d), and L represents the body length of the experimental fish (cm).

#### 2.4.2. Determination of the Chroma Value

The L*, a*, and b* defined by the CIE are used to represent the body color state of the fish. L* represents brightness (luminance). The value of a* represents redness (+a* indicating a bias toward red, −a* indicating a bias toward green), while the value of b* represents yellowness (positive values indicating a bias toward yellow, while negative values indicating a bias toward blue) [1,19]. The L*, a*, and b* values of 6 fish were randomly measured in each replicate group using a CR-400 colorimeter (Konica-Minolta, Tokyo, Japan), which was calibrated with a white board before use. The water on the surface of the fish body was then absorbed with absorbent paper. Next, the probe of the colorimeter was pressed against the red area below the dorsal fin of the fish body and above the side line, and the reading was scanned and recorded.

#### 2.4.3. Determination of Carotenoid Content

The extraction and determination of the carotenoids were conducted according to the method described by Yang et al. (2012) [20]. The computation formula for the carotenoid content is as follows:

*S* = (*A* × K × *V*)/(*E* × *G*)

In the formula, *S* represents the carotenoid content (mg/kg); *A* represents the absorbance value; K is a constant (10,000); *V* represents the volume of extracted liquid (mL); *E* represents the absorption coefficient (2500); and *G* represents the sample weight (g).

#### 2.4.4. Preparation of Hematoxylin–Eosin (HE)-Stained Tissue Sections

The liver tissue samples were fixed with 4% paraformaldehyde and dehydrated step by step using a gradient of alcohol (70%, 80%, 90%, 95%, and 100%). After being treated with half xylene, xylene I, and xylene II to achieve transparency, the samples were embedded in paraffin (with a thickness of 5 μm), sectioned, stained with HE dye, sealed with neutral gum, and observed and photographed under a ZEISS microscope (Axio Scope. A1, Oberkochen, Germany).

#### 2.4.5. Determination of Liver Biochemical Indexes

The activities of lysozyme (LZM), alkaline phosphatase (AKP), and superoxide dismutase (SOD) were quantitatively measured using the lysozyme assay kit (A050-1-1, turbidimetry), alkaline phosphatase assay kit (A059-2-2, microplate method), and superoxide dismutase (SOD) assay kit (A001-3-2, WST-1 method). All liver biochemical indexes were determined using commercial assay kits (Nanjing Jiancheng Bioengineering Institute, Nanjing, China) according to the manufacturer’s instructions, respectively.

#### 2.4.6. Fluorescence qRT-PCR Assay of IGF-1, LPL, and TYR

The total RNA extraction and reverse transcription were performed following the instructions provided with the Tissue RNA Kit (OMEGA, Tarzana, CA, USA) for scale, skin, liver, and muscle samples. The extracted RNA was dissolved in diethyl pyrocarbonate (DEPC) water and assessed for the integrity, purity, and concentration using a 1% agarose gel and a Multifunctional Enzyme Labeler (BioTek Cytation 5, Winooski, VT, USA). The resulting cDNA samples were stored at −20 °C for PCR amplification. The first strand of cDNA synthesis was performed using the PrimeScript^TM^ II 1st Strand cDNA Synthesis Kit (TaKaRa, Shiga, Japan), followed by storage at −20 °C.

The expression levels of the insulin-like growth factor 1 (*IGF-1*), lipoprotein lipase (*LPL*), and tyrosinase (*TYR*) genes were detected using real-time fluorescent quantitative PCR. The primers were designed using Premier 5.0 (Premier Biosoft, San Francisco, CA, USA) software based on the conserved sequences of koi genes in GenBank (Table 2). All the primers were synthesized by Guangzhou Aiji Biotechnology Co., Ltd. (Guangzhou, China).

The cDNA of each group was selected as the template for the RT-PCR amplification, and the relative gene expression was calculated 2^−ΔΔCt^ using β-actin as the internal parameter [21].

### 2.5. Data Processing and Analysis

The experimental data were expressed as the mean ± standard deviation (mean ± SD). Since the experimental design involved a single factor (i.e., whole-fat or defatted Antarctic krill powder), the statistical analyses were performed using SPSS version 26.0 via a one-way analysis of variance (ANOVA). A significance level of *p* < 0.05 was set as the threshold for determining statistical significance.

## 3. Results

### 3.1. Effects of Replacing Fish Meal with Whole-Fat or Defatted Antarctic Krill Meal on the Growth of Red–White Koi Carp

The SGR of the red–white koi exhibited a gradually increasing trend as the level of Antarctic krill meal increased instead of fish meal, as shown in Table 3. Meanwhile, the viscerosomatic ratio and HSI showed a decreasing trend. There were no significant differences in the specific growth rate and weight gain rate between the whole-fat group and the defatted group (*p* > 0.05). The HSI in the W30 group was significantly lower than that in the D30 group (*p* < 0.05), while the SR showed a significant decreasing trend in the whole-fat group (*p* < 0.05) but not in the defatted group (*p* > 0.05). The fatness of the red–white koi decreased gradually in both groups, but there was no significant difference between the defatted groups (*p* > 0.05). The Antarctic krill meal had significant effects on the HSI, VSI, and CF, and it exhibited a significant interaction with the fish meal level; however, the fish meal level had no significant effect on any of the indicators. To test the interactive effects, the influence of each factor on the observed indicators was separately examined.

### 3.2. Effects of Replacing Fish Meal with Whole-Fat or Defatted Antarctic Krill Powder on Body Color of Red–White Koi Carp

The L* value of the red region on the body surface of the red–white koi showed no significant difference among all the groups with increasing levels of krill powder substitution (*p* > 0.05), as indicated in Table 4. The W30 group exhibited the highest a* value, and the a* values of the W30, D30, and W20 groups were significantly higher than those of the other groups (*p* < 0.05). Furthermore, the b* values of the W20 and W30 groups were significantly higher than those of the other groups (*p* < 0.05). The levels of fish meal and Antarctic krill meal did not show significant effects on any of the indicators, and there was no significant interaction between them.

**Figure 1:**
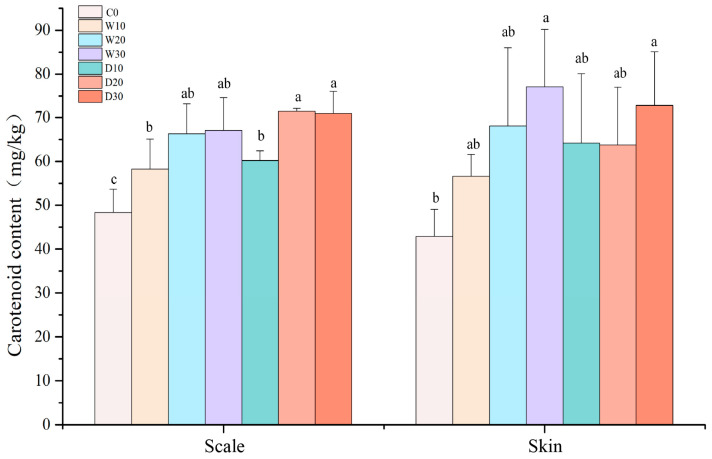
Effects of replacing fish meal with whole-fat or defatted Antarctic krill meal on the carotenoid content in the skin and scales of red–white koi carp. Note: Data without common superscript letters show significant differences between groups (*p* < 0.05).

The levels of carotenoid in the scales and skin of the red–white koi showed a significant increase when fish meal was substituted with varying amounts of whole-fat or defatted krill powder, as depicted in Figure 1 (*p* < 0.05). With an increasing proportion of krill powder replacement, there was a gradual rise in the carotenoid levels found in both the scales and skin of the red–white koi. There were no notable differences observed regarding the carotenoid content among all the experimental groups (*p* > 0.05).

### 3.3. Effects of Replacing Fish Meal with Whole-Fat or Defatted Antarctic Krill Powder on Liver of Red–White Koi Carp

**Figure 2:**
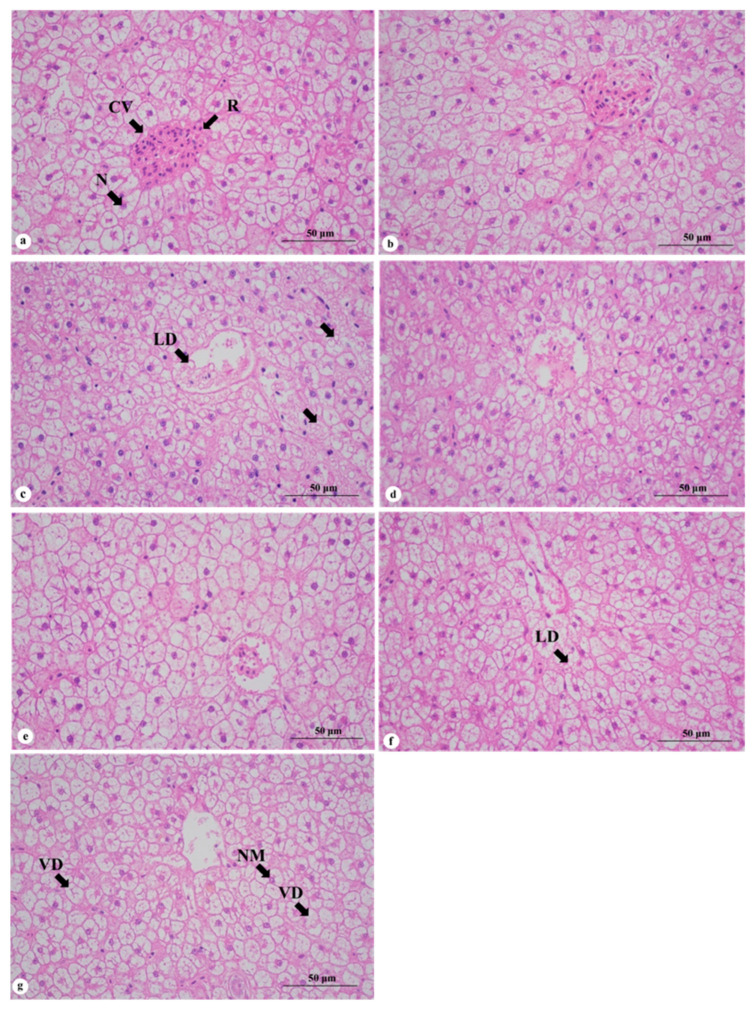
Effects of replacing fish meal with Antarctic krill meal on the liver structure of koi carp (400×). Note: (**a**) C0 group; (**b**) W10 group; (**c**) W20 group; (**d**) W30 group; (**e**) D10 group; (**f**) D20 group; and (**g**) D30 group. N: nucleus; NM: nucleus migration; VD: vacuolar degeneration: CV: central veins: R: red blood cell; LD: lipid droplet.

The liver cells in each group exhibited a normal morphology with clear boundaries and a uniform arrangement, as shown in Figure 2. Additionally, some cells displayed nuclear migration and vacuolation. There were no significant differences in the liver tissue between the control group and the W10 and D10 groups. In the high-fat diet groups, both the W20 and W30 groups showed a decrease in the hepatocyte volume compared to the W10 group. Moreover, an abundance of lipid droplets was evident in the W20 group, while their quantity decreased in the W30 group. Within the defatted groups, there were no significant differences found in the hepatocyte volume among the D10, D20, and D30 groups; however, both the D20 and D30 groups exhibited an increase in the lipid droplet count along with an elevated occurrence of vacuolar degeneration within the hepatocytes.

### 3.4. Effects of Substituting Fish Meal with Whole-Fat or Defatted Antarctic Krill Meal on Non-Specific Immune Indexes of Red–White Koi Carp

The liver AKP activity of the koi exhibited a significant decrease in both the D20 and D30 groups compared to the other groups, as shown in Table 5 (*p* < 0.05). Moreover, an increase in the krill powder substitution level was associated with a gradual rise in the liver LZM activity and a significant decrease in the SOD activity (*p* < 0.05). It is worth noting that both the LZM and SOD activities were significantly higher in the experimental groups than those observed in the control group (*p* < 0.05), especially for the LZM activities, which were notably elevated, specifically within the D30 group compared to the other groups (*p* < 0.05). Additionally, the SOD activity was higher in the defatted groups than that observed within the whole-fat groups; among all the defatted samples tested, it reached its peak value within the D10 group (*p* < 0.05). The Antarctic krill meal powder significantly influenced the AKP, LZM, and SOD activities, with a notable interaction effect between the levels of Antarctic krill meal powder and fish meal. Additionally, the fish meal level exhibited a significant regulatory effect on the SOD activity.

### 3.5. Effects of Replacing Fish Meal with Whole-Fat or Defatted Antarctic Krill Meal on the Expression Levels of IGF-1, LPL and TYR in the Liver and Muscle Tissue of Red–White Koi Carp

**Figure 3:**
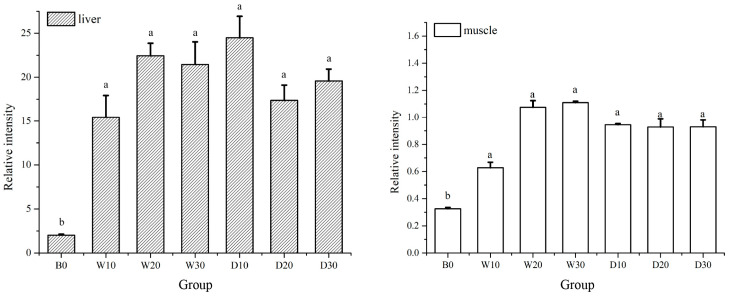
Effects of replacing fish meal with whole-fat or defatted Antarctic krill meal on the relative expression of IGF-1 in the liver and muscle of red–white koi carp. Note: Data without common superscript letters show significant differences between groups (*p* < 0.05).

The results showed a relatively high expression level of *IGF-1* in the liver tissues of the red–white koi carp (Figure 3). Replacing fish meal with krill meal gradient significantly enhanced the *IGF-1* expression in the liver of the red–white koi carp compared to the control group (*p* < 0.05), without any significant difference observed among the experimental groups (*p* > 0.05). Additionally, a similar pattern was overserved regarding the expression level of *IGF-1* in the muscle tissue.

**Figure 4:**
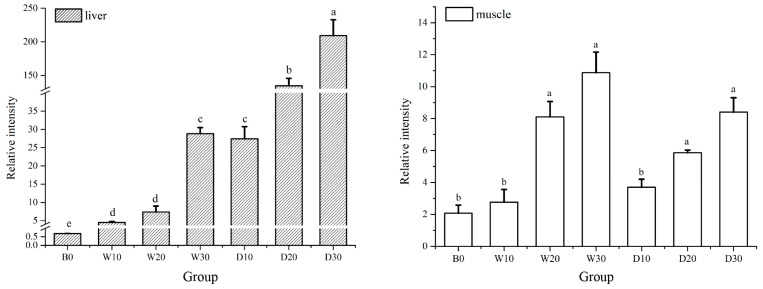
Effects of replacing fish meal with whole-fat or defatted Antarctic krill meal on the relative expression of *LPL* in the liver and muscle of red–white koi carp. Note: Data without common superscript letters show significant differences between groups (*p* < 0.05).

The expression levels of *LPL* were positively correlated with the replacement difference in both the liver and muscle tissues of the koi carp, as shown in Figure 4. In liver, the relative expression level of *LPL* was significantly lower in C0 compared to the other groups (*p* < 0.05), there was no significant difference between the W10 and W20 groups (*p* > 0.05), but there was a significant level between the W30 and D20 groups (*p* < 0.05), with an even higher expression level observed in the D30 group (*p* < 0.05). In the muscle tissue, all the experimental groups (W20, W30, D20, and D30) exhibited significantly higher levels of *LPL* expression compared to the other groups; however, there were no significant differences among these four groups (*p* > 0.05). Additionally, it is worth noting that the relative *LPL* expression in the liver of the defatted groups exceeded that of the whole-fat groups.

**Figure 5:**
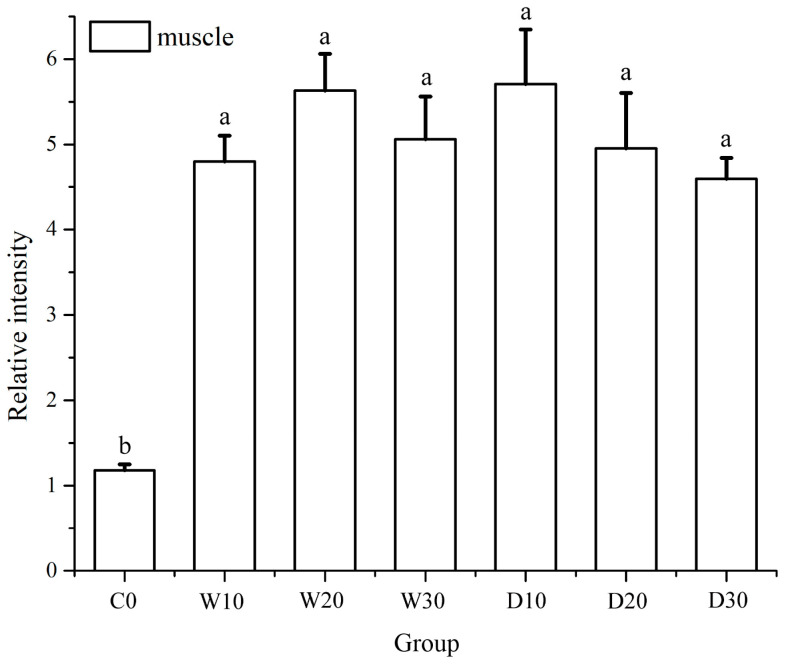
Effects of replacing fish meal with whole-fat or defatted Antarctic krill meal on the relative expression of TYR in the muscle of red–white koi carp. Note: Data without common superscript letters show significant differences between groups (*p* < 0.05).

The results revealed an absence of *TYR* expression in the liver of the red–white koi, while exhibiting a low level of expression in the muscle (Figure 5). Notably, the C0 group displayed significantly lower muscle expression compared to the other groups (*p* < 0.05), whereas no significant differences were observed among the remaining groups (*p* > 0.05).

## 4. Discussion

### 4.1. Effects of Replacing Fish Meal with Whole-Fat or Defatted Antarctic Krill Powder on Growth Performance of Red–White Koi Carp

Antarctic krill meal, with its rich nutrient content and vast oceanic biomass, holds great potential as a novel animal protein source that could fully replace fish meal [8,11]. Incorporating an appropriate amount of Antarctic krill meal into the diets of fish species such as Atlantic cod (*Gadus morhua*), *Litopenaeus Vannamei* shrimp, and *Cynoglossus semilaevis* flounder can enhance their growth performance without significantly affecting the survival rate or specific growth rate of *Atlantic salmon*, rainbow trout (*O. mykiss*), Atlantic halibut (*Hippoglossus hippoglossus flounder*), and turbot (*Scophthalmus maximus*) [15,22]. In this experiment, there was no significant difference in the survival rate among all the groups. The SGR of the red–white koi in the experimental groups was significantly improved. However, there was no significant difference between the groups with different substitution levels. This indicates that replacing fish meal with Antarctic krill meal could significantly enhance the growth performance of red–white koi. Nevertheless, the effect of using whole-fat or defatted krill meal on growth was not significant. The substitution level and effect of Antarctic krill powder in the feed for different fish species vary due to the specific species, diet, and experimental environment factors. With an increase in the substitution level of Antarctic krill powder, there was a gradual decrease in fatness. It is speculated that excessive consumption of Antarctic krill powder affected the fish’s ability to digest fat, resulting in reduced fat content [23].

The HSI and VSI are important indices for evaluating the health and quality of fish. The HSI coefficient reflects the amount of fat deposited in the liver and pancreas, while the VSI coefficient indicates whether there is congestion, enlargement, disease, atrophy or other degenerative changes in the internal organs [24]. In the results of this experiment, the HSI initially decreased and subsequently increased in the whole-fat groups, whereas it showed a gradually decreasing trend in the defatted groups. The HSI index was higher in the defatted groups than that in the whole-fat groups, indicating lower liver fat deposition compared to the whole-fat groups and compared to the nonfat group. This could be attributed to the Antarctic krill oil present in the whole-fat Antarctic krill powder since it has been shown to reduce the total cholesterol, triglycerides, and low-density lipoprotein index [25]. Consequently, a reduction in liver fat deposition was observed in the whole-fat groups. The significant decrease of the VSI in the whole-fat groups may be attributed to a decrease in the liver fat content, leading to a reduced total visceral mass weight and a significant decrease in the VSI index.

### 4.2. Effect of Replacing Fish Meal with Whole-Fat or Defatted Antarctic Krill Powder on Body Color of Red–White Koi Carp

Fish, like other vertebrates, are unable to synthesize carotenoids from scratch; therefore, their skin color depends on the absorption and deposition of carotenoids obtained from food [26]. Antarctic krill powder is rich in natural astaxanthin, with a concentration of about 180 mg/kg. Astaxanthin is the end product of carotenoid synthesis and can be directly stored and deposited in fish tissues. It can also bind non-specifically with myoglobin. Compared to other carotenoids, astaxanthin is more easily absorbed and accumulated by fish [8]. Therefore, Antarctic krill powder also has the effect of increasing the bright pigment in aquatic animals. The use of Antarctic krill meal instead of fish meal can enhance the redness of Atlantic cod skin near the lateral line scale, resulting in whiter skin on the fish body and a stronger yellow tone [27]. When fed only the compound feed, the body color of Japanese Amberjack (*Seriola quinqueradiata*) turned black, losing the blue–green luster of wild fish, and the yellow color disappeared near the measurement line. Adding 2% Antarctic krill oil to the compound feed resulted in an improvement in the body color change [28]. In the results of this experiment, there was a significant increase in the carotenoid content in both the scales and skin of red–white koi in the experimental groups compared to that in the control group when fish meal was replaced with Antarctic krill meal. As the level of krill powder substitution increased, there was an upward trend in the carotenoid content in both the red–white koi scales and skin; however, there were no significant differences among the different substitution levels. Additionally, there were no significant differences in the carotenoid content between the whole-fat groups and defatted groups.

The L* values represent the brightness, the a* values represent the redness, and the b* values represent the yellowness among the chromaticity values that represent objects. The levels of these values are positively correlated with the intensity of the chromaticity [19]. Substituting fish meal with krill meal in the feed of an Atlantic cod can significantly increase its body surface’s a* and b* values. By adding Antarctic krill powder to the feed of cultured red porgy (*Pagrus pagrus*), it is possible to achieve the same red body color as wild red porgy [26]. In this experiment’s results, groups W20, W30, and D30 exhibited significantly higher a* and b* values on their body surfaces compared to the other groups. Additionally, when fish meal was replaced with whole-fat or defatted krill powder, groups W20 and W30 showed significantly higher b* values than the other groups. The results showed that using Antarctic krill meal instead of fish meal could improve both the a* and b* values on the body surface of red–white koi to some extent, enhancing the color richness of their bodies. When compared with the nonfat groups, the red–white koi in the whole-fat groups exhibited relatively higher a* and b* values on their body surface. This difference could be attributed to the presence of Antarctic krill oil in the whole-fat Antarctic krill powder, which contains 40–5000 mg/kg astaxanthin [11,29]. In contrast, the nonfat Antarctic krill powder lacks Antarctic krill oil, thereby weakening its coloring ability.

### 4.3. Effect of Replacing Fish Meal with Whole-Fat or Defatted Antarctic Krill Meal on Liver of Red–White Koi Carp

The liver is the main digestive organ of fish, responsible for absorbing nutrients, secreting digestive enzymes, and playing a role in transporting and storing nutrients. Adding 2–4% Antarctic krill powder to the diet has certain positive effects on the production of digestive enzyme, nutrient absorption, and fat and glycogen storage functions in *L. vannamei* [30]. Yoshitomi conducted a study on the effect of replacing fish meal with an Antarctic krill meal gradient on rainbow trout. The results indicated that there were no histopathological changes in the liver structure of the rainbow trout among all the groups, suggesting that the presence of fluorine in the Antarctic krill meal did not affect the liver tissue of the rainbow trout [7]. In this study, when the level of fish meal replaced by Antarctic krill meal was at 10%, there was no significant difference between the liver tissue of the red–white koi carp and that of the control group. However, when the level of krill meal replaced by Antarctic krill meal was increased to 20%, the liver cells in the whole-fat groups became smaller and showed an increase in fat droplets. The number of fat droplets in the high-fat groups exceeded that in the defatted groups, and there was a positive correlation between the volume of liver cells and the liver fat content. The decrease in hepatocytes and the increase in lipid droplets indicated an elevation in liver lipid metabolism and glycogen consumption. These results were consistent with the results of the HSI. The number of lipid droplets in the hepatocytes was higher in the whole-fat groups compared to the nonfat groups, possibly due to the inclusion of Antarctic krill oil in the whole-fat Antarctic krill powder, which can increase the body’s high-density lipoprotein index. High-density lipoprotein is capable of extracting cholesterol from the cell membrane and transporting it back for liver metabolism for the aging cell membrane and plasma found in peripheral tissues [25]. It is speculated that the Antarctic krill oil contained in the whole krill powder can improve the liver’s fat metabolism to some extent. The D30 group showed an increase in the vacuolar degeneration of hepatocytes, indicating a trend toward liver injury. It is hypothesized that defatted krill powder contains a high proportion of fluorine and chitin, while red–white koi are freshwater fish that need to continuously absorb minerals from fresh water to meet their body’s needs. Consequently, they are prone to accumulating a high proportion of fluorine in their bodies. When excessive amounts of defatted krill powder are added, it leads to the increased accumulation of fluorine in the bodies of red–white koi carp, resulting in liver injury.

### 4.4. Effect of Substituting Fish Meal with Whole-Fat or Defatted Antarctic Krill Meal on Non-Specific Immune Indexes of Red–White Koi Carp

LZM, as the main indicator of non-specific immunity, can hydrolyze and release acetyl aminoglycans from the mucopeptides in the cell walls, thereby destroying and eliminating foreign bodies that invade the body and enhancing the body’s defense function [31]. In this study, the LZM activity was significantly increased in each experimental group compared to the control group, indicating an enhanced ability of the body to defend against bacterial invasion. AKP is a zinc-containing phosphate monoester hydrolase with low substrate specificity. It mainly exists in the superficial and striate margin of fish anterior intestinal epithelial cells, directly participating in the transfer and metabolism of phosphate groups in vivo. It contributes to the absorption of intestinal epithelial cells and is positively correlated with the absorption of lipids, glucose, calcium, and inorganic phosphorus. Additionally, it plays an important role in bone mineralization in aquatic animals [32]. There was no significant difference observed in the AKP activity among all the groups fed with the whole-fat krill powder, while the AKP activity significantly decreased in the 20% and 30% defatted groups. This suggests that when the defatted krill powder reached replacement levels of 20% and 30%, the red–white koi experienced decreased nutritional metabolism levels. However, the increase in the replacement level did not affect the nutritional metabolism level of the whole-fat groups. It is speculated that excessive levels of added defatted Antarctic krill powder, which contains high amounts of fluorine and chitin, may have adverse effects on the body [33].

Typically, a fish’s antioxidant defense mechanism regulates the activity of antioxidant enzymes to prevent the production of reactive oxygen species [34]. SOD, a crucial antioxidant enzyme employed by organisms for eliminating oxygen free radicals, plays a vital role in maintaining low levels of oxidative metabolism and preventing oxidative damage within the body [35]. In this experiment, the SOD activity of the experimental groups was significantly higher than that of the control group, indicating that incorporating Antarctic krill powder into the diet played a significant role in enhancing the antioxidant capacity of the red–white koi. However, with the increase in the replacement level of Antarctic krill powder, the SOD activity significantly decreased. This may be attributed to the fact that Antarctic krill meal is rich in astaxanthin, a potent antioxidant that specifically eliminates superoxide free radicals. As astaxanthin effectively reduces free radicals, the substrate for SOD activity decreases, leading to a decline in the SOD enzyme activity. Additionally, the defatted group exhibited higher SOD activity compared to the full-fat group. This could be due to the presence of abundant unsaturated fatty acids in full-fat Antarctic krill meal, which are more susceptible to oxidation and may consequently affect its functionality.

### 4.5. Effects of Replacing Fish Meal with Whole-Fat or Defatted Antarctic Krill Powder on the Expression of IGF-1, LPL and TYR in the Liver and Muscle Tissue of Red–White Koi Carp

Like other species, the expression of the *IGF-1* gene in fish is influenced by their nutritional status, and the nutrition levels also impact the *IGF-1* expression [36]. Detecting the level of *IGF*-*1* expression serves as a crucial indicator of the fish growth rate and provides an essential basis for evaluating the effects of different feed formulas [37]. Previous studies have demonstrated that incorporating Antarctic krill oil into diets effectively promotes longitudinal bone growth in male baby rats [38]. Additionally, Antarctic krill oil can regulate the secretion of genes related to the *IGF-1* pathway, thereby promoting the growth and development of young rats [38]. Furthermore, supplementation with 100 g/kg krill hydrolysate in compound feed improves Turbot’s growth performance and up-regulate the expression of *IGF-1*, *PepT1,* and *NPY* [39]. In this experiment, the addition of Antarctic krill powder significantly increased the expression level of *IGF*-*1* in the koi carp. There was no significant difference in the *IGF-1* expression between the whole-fat groups and the defatted groups. The liver exhibited higher levels of *IGF-1* expression compared to the muscle, and the expression levels of *IGF-1* were observed to change in synchrony with the growth performance of the koi carp. These findings suggest that substituting fish meal with Antarctic krill meal can enhance the growth performance of koi carp by regulating the *IGF-1* expression in both the liver and muscle.

LPL is an enzyme that catalyzes the hydrolysis of protein-associated triglycerides, thereby facilitating the breakdown of chylomicron and triglycerides carried by VLDL in the bloodstream into glycerol and fatty acids for subsequent storage and utilization in various tissues. The elevation of dietary fat levels has been shown to suppress the activity and gene expression of fatty acid synthetase while concurrently enhancing the activity and gene expression of *LPL* [40,41]. Conversely, Liang et al. reported that there was no significant increase in the *LPL* expression levels in the hepatopancreas of red sea bream (*Pagrosomus major*) when exposed to varying dietary fat levels [42]. In this experiment, the expression level of the *LPL* gene in the liver was higher than that in the muscle, which is consistent with previous research on *Cyprinus carpiovar* var. Jian [43], *Sparus aurata* [44], and rainbow trout [45]. As the substitution level of the krill powder increased, there was a gradual increase in the relative expression of *LPL* in both the liver and muscle tissues of the koi. The Antarctic krill meal is rich in N-3 polyunsaturated fatty acids (PUFAs) bound in phospholipid form, particularly docosahexaenoic acid (DHA) and eicosapentaenoic acid (EPA). A substantial proportion of these PUFAs are present as phosphatidylcholine. In contrast, the feed for the control group contained a higher proportion of fish oil, which has a greater fat content. The fatty acids in fish oil predominantly exist in triglyceride form [46]. Prior studies have demonstrated that the structural differences between phospholipids and triglycerides lead to more efficient accumulation of DHA and EPA in phospholipid form within the brains, livers, and other organs of both animals and humans [47]. This may explain why the relative expression levels of *LPL* were the lowest in the liver and muscle tissues of both red and white goldfish in the control group. It appears that the regulatory effects of the morphological characteristics and compositional quality of dietary fats on the *LPL* activity surpass the influence of the fat content alone. The relative expression level of *LPL* in the liver was higher in the defatted groups compared to the whole-fat groups. The krill oil had a lipid-lowering effect, resulting in a lower fat content in the koi from the whole-fat groups relative to those from the defatted groups. The *LPL* enzyme regulated by the *LPL* gene can catalyze triglycerides present in chylomicron particles and VLDL, with its gene expression level being positively correlated with the chylomicron particles, VLDLs, and triglyceride contents [13]. Therefore, the relative expression of *LPL* in the liver was lower in the whole-fat group.

*TYR* plays a crucial role in melanin formation and serves as one of the most crucial rate-limiting enzymes in the animal melanin synthesis pathway, with its expression level determining the type and speed of melanin production [48]. The tissue-specific expression patterns of *TYR* vary among fish species; for instance, it is expressed in the eyes and skin of *Oryzias latipes* but not in the liver [49]. In a study on orange diplocercids conducted by Jiang Yanling, it was observed that during the process of the color fading from black to gray to bright yellow, there was a gradual decrease in the *TYR* expression. This finding suggests a potential correlation between this decline and an increase in red pigment cells and yellow pigment cells, as well as alterations in both the number and distribution ratio of melanocytes [50]. The results of this experiment showed that *TYR* was not expressed in the livers of the red–white koi, but it had a low expression level in the muscles. The expression level of *TYR* was significantly higher in the experimental groups compared to the control group, which can be speculated to be due to the astaxanthin present in Antarctic krill powder promoting pigmentation. As liver and muscle tissues were used for this experiment, the differences observed might be associated with the fish species and tissue sampling [36].

## 5. Conclusions

The findings of this experiment demonstrate that substituting a certain amount of whole-fat or defatted Antarctic krill meal for fish meal can enhance the growth performance, immune response, and body coloration of koi carp. No significant differences were observed between the two types of krill powder in terms of the specific growth rate, weight gain rate, and body coloration in koi carp. Defatted krill powder exhibited superior effects on enhancing the immune capacity of koi carp. However, when the replacement level exceeded 20%, it resulted in liver damage, whereas whole-fat krill powder was found to be more beneficial for liver health. The recommended replacement levels are 10% to 20% for defatted krill powder and 20% to 30% for whole-fat krill powder.

## Figures and Tables

**Table 1:** Experimental feed composition and nutritional level (air-dry basis).

Ingredient (g/kg)	Group						
	C0	W10	W20	W30	D10	D20	D30
Wheat flour	220.00	220.00	220.00	220.00	220.00	220.00	220.00
Imported fish meal	500.00	400.00	300.00	200.00	400.00	300.00	200.00
Soy Protein	110.00	110.00	110.00	110.00	110.00	110.00	110.00
Whole-fat Antarctic krill meal	0.00	100.00	200.00	300.00	0.00	0.00	0.00
Defatted Antarctic krill meal	0.00	0.00	0.00	0.00	100.00	200.00	300.00
Yeast	50.00	50.00	50.00	50.00	50.00	50.00	50.00
Seaweed meal	20.00	20.00	20.00	20.00	20.00	20.00	20.00
Soybean oil	50.00	50.00	50.00	50.00	50.00	50.00	50.00
Ca (H_2_PO_4_)_2_	20.00	20.00	20.00	20.00	20.00	20.00	20.00
Choline chloride	5.00	5.00	5.00	5.00	5.00	5.00	5.00
Amino acid ^1^	10.00	10.00	10.00	10.00	10.00	10.00	10.00
Vitamin premix ^2^	5.00	5.00	5.00	5.00	5.00	5.00	5.00
Mineral premix ^3^	10.00	10.00	10.00	10.00	10.00	10.00	10.00
Total	1000	1000	1000	1000	1000	1000	1000
Nutrient levels (%)							
Crude protein	48.19	48.09	47.99	47.69	48.60	49.00	49.20
Crude fat	8.75	8.70	8.65	8.85	8.20	7.65	7.35

Note: ^1^ Lysine and methionine 1:1. ^2^ Vitamin A, 1,100,000 IU; vitamin D3, 320,000 IU; nicotinic acid, 7800 mg; vitamin E, 2500 mg; vitamin B1, 1000 mg; biotin, 8 mg; vitamin B2, 2000 mg; vitamin B6, 1000 mg; folic acid, 400 mg; vitamin B12, 125 mg; vitamin C, 18,000 mg; enzyme, 1000 mg; K, 1.1%; NaCl, 4.5%; methionine, 400 mg; moisture, ≤10%. ^3^ Mineral premix provided the following per 1 kg of the diet: P, 400 mg; Fe, 70 g; Cu, 200 mg; Zn, 30 mg; Mn, 20 mg; Mg, 250 mg; I, 30 mg; Co, 10 mg; Se, 10 mg; the carrier was zeolite powder. ME was a calculated value, while the others were measured values.

**Table 2:** Primer sequences for the real-time fluorescence quantification PCR.

Primers	Primer Sequences (5′→3′)
*β-actin* F	TGCAAAGCCGGATTCGCTGG
*β-actin* R	AGTTGGTGACAATACCGTGC
*IGF-1* F	GCAGTGTACCATGCGCTGTCT
*IGF-1* R	GAAATAAAAGCCCCTGTCTCCA
*TYR* F	GCCCGTCCTCGGTGTTCTCC
*TYR* R	GGTTTGGGTCGTGGTTTCCT
*LPL* F	CGATTGARCCARCGAGTG
*LPL* R	CTTGTTGCARCGRTTCTT

**Table 3:** Effects of replacing fish meal with whole-fat or defatted Antarctic krill meal on the growth performance of red–white koi carp.

Group				Item				
	Initial Weight/g	Final Weight/g	SR/%	SGR (%d)	WGR/%	HSI/%	VSI/%	CF (g/cm^3^)
C0	13.87 ± 0.65 ^a^	61.23 ± 12.46 ^a^	100.00 ± 0.00	2.44 ± 0.24 ^b^	335.88 ± 62.58 ^b^	2.78 ± 0.53 ^a^	8.79 ± 0.85 ^a^	3.41 ± 0.18 ^a^
W10	13.63 ± 0.58 ^a^	65.33 ± 8.80 ^a^	100.00 ± 0.00	2.57 ± 0.27 ^a^	372.74 ± 74.49 ^a^	3.08 ± 0.46 ^a^	9.18 ± 0.44 ^a^	3.43 ± 0.32 ^a^
W20	13.98 ± 1.42 ^a^	75.48 ± 8.17 ^a^	100.00 ± 0.00	2.79 ± 0.18 ^a^	435.23 ± 55.64 ^a^	2.23 ± 0.11 ^d^	8.27 ± 0.74 ^b^	3.07 ± 0.19 ^c^
W30	14.03 ± 1.27 ^a^	74.99 ± 6.57 ^a^	100.00 ± 0.00	2.77 ± 0.23 ^a^	430.20 ± 73.10 ^a^	2.41 ± 0.28 ^c^	5.68 ± 0.35 ^c^	3.07 ± 0.17 ^c^
D10	13.04 ± 1.19 ^a^	70.36 ± 9.64 ^a^	100.00 ± 0.00	2.79 ± 0.18 ^a^	435.12 ± 58.15 ^a^	3.18 ± 0.49 ^a^	6.31 ± 0.68 ^c^	3.18 ± 0.22 ^b^
D20	13.11 ± 1.47 ^a^	66.17 ± 6.46 ^a^	100.00 ± 0.00	2.67 ± 0.23 ^a^	399.46 ± 66.97 ^a^	2.85 ± 0.38 ^a^	5.86 ± 0.50 ^c^	3.24 ± 0.27 ^b^
D30	13.50 ± 0.56 ^a^	79.68 ± 12.50 ^a^	100.00 ± 0.00	2.96 ± 0.04 ^a^	489.04 ± 14.16 ^a^	2.68 ± 0.25 ^b^	6.12 ± 0.64 ^c^	3.14 ± 0.21 ^b^

Note: SR: survival rate, SGR: special gain rate, WGR: weight gain rate, HSI: hepatosomatic index, VSI: viscerosomatic index. Values in the same column with different superscripts are significantly different (*p* < 0.05).

**Table 4:** Effects of replacing fish meal with whole-fat or defatted Antarctic krill meal on the L*, a*, b* values of the body surface of red–white koi carp.

Group	L*	a*	b*
C0	58.46 ± 4.97	6.42 ± 1.85 ^b^	25.10 ± 6.72 ^c^
W10	58.90 ± 3.40	5.49 ± 1.79 ^b^	25.00 ± 4.87 ^c^
W20	58.42 ± 3.78	7.98 ± 2.61 ^a^	29.05 ± 6.14 ^a^
W30	56.45 ± 3.38	8.85 ± 2.67 ^a^	30.96 ± 6.15 ^a^
D10	58.36 ± 3.28	5.94 ± 2.27 ^b^	24.25 ± 5.58 ^c^
D20	58.91 ± 4.47	5.75 ± 2.45 ^b^	26.83 ± 5.71 ^b^
D30	57.95 ± 8.40	7.75 ± 2.21 ^a^	27.50 ± 6.18 ^b^

Note: Data without common superscript letters in the same column show significant differences between groups (*p* < 0.05).

**Table 5:** Effects of replacing fish meal with whole-fat or defatted Antarctic krill meal on the liver non-specific immunity indexes of red–white koi carp.

Group	Item		
	AKP (U/mg Protein)	LZM (U/mg Protein)	SOD (U/mg Protein)
C0	10.03 ± 2.09 ^a^	41.07 ± 7.27 ^d^	1.79 ± 0.56 ^d^
W10	10.53 ± 1.72 ^a^	58.89 ± 6.61 ^c^	8.29 ± 0.13 ^b^
W20	11.42 ± 2.51 ^a^	55.13 ± 10.20 ^c^	4.27 ± 1.70 ^b^
W30	13.06 ± 2.09 ^a^	63.91 ± 10.90 ^c^	2.76 ± 1.03 ^c^
D10	12.27 ± 2.13 ^a^	62.05 ± 6.70 ^c^	31.02 ± 9.52 ^a^
D20	7.98 ± 1.73 ^b^	84.78 ± 3.70 ^b^	11.30 ± 4.50 ^b^
D30	7.21 ± 2.19 ^b^	120.83 ± 4.90 ^a^	9.50 ± 2.99 ^b^

Note: AKP: alkaline phosphatase, SOD: superoxide dismutase, LZM: lysozyme. Data without common superscript letters show significant differences between groups (*p* < 0.05).

## Data Availability

The original contributions presented in the study are included in the article; further inquiries can be directed to the corresponding author.
